# Detoxification of Deoxynivalenol via Glycosylation Represents Novel Insights on Antagonistic Activities of *Trichoderma* when Confronted with *Fusarium graminearum*

**DOI:** 10.3390/toxins8110335

**Published:** 2016-11-15

**Authors:** Ye Tian, Yanglan Tan, Na Liu, Zheng Yan, Yucai Liao, Jie Chen, Sarah de Saeger, Hua Yang, Qiaoyan Zhang, Aibo Wu

**Affiliations:** 1 SIBS-UGENT-SJTU Joint Laboratory of Mycotoxin Research, Key Laboratory of Food Safety Research, Institute for Nutritional Sciences, Shanghai Institutes for Biological Sciences, Chinese Academy of Sciences, University of Chinese Academy of Sciences, 294 Taiyuan Road, Shanghai 200031, China; tianye@sibs.ac.cn (Y.T.); yltan@sibs.ac.cn (Y.T.); liuna@sibs.ac.cn (N.L.); zyan@sibs.ac.cn (Z.Y.); 2 College of Plant Science and Technology, Huazhong Agricultural University, Wuhan 430070, China; yucailiao@mail.hzau.edu.cn; 3 Department of Resources and Environment Sciences, School of Agriculture and Biology, Shanghai Jiaotong University, 800 Dongchuan Road, Shanghai 200240, China; jiechen59@sjtu.edu.cn; 4 Laboratory of Food Analysis, Department of Bioanalysis, Faculty of Pharmaceutical Sciences, Ghent University, Ottergemsesteenweg 460, B-9000 Ghent, Belgium; Sarah.DeSaeger@UGent.be; 5 State Key Laboratory Breeding Base for Zhejiang Sustainable Pest and Disease Control, Institute of Quality and Standard for Agro-Products, Zhejiang Academy of Agricultural Sciences, Hangzhou 310021, China; yanghua806@hotmail.com (H.Y.); yanyan0014@163.com (Q.Z.)

**Keywords:** mycotoxin, toxigenic *Fusarium*, biological control, *Trichoderma*, modified mycotoxin

## Abstract

Deoxynivalenol (DON) is a mycotoxin mainly produced by the *Fusarium graminearum* complex, which are important phytopathogens that can infect crops and lead to a serious disease called *Fusarium* head blight (FHB). As the most common B type trichothecene mycotoxin, DON has toxic effects on animals and humans, which poses a risk to food security. Thus, efforts have been devoted to control DON contamination in different ways. Management of DON production by *Trichoderma* strains as a biological control-based strategy has drawn great attention recently. In our study, eight selected *Trichoderma* strains were evaluated for their antagonistic activities on *F. graminearum* by dual culture on potato dextrose agar (PDA) medium. As potential antagonists, *Trichoderma* strains showed prominent inhibitory effects on mycelial growth and mycotoxin production of *F. graminearum*. In addition, the modified mycotoxin deoxynivalenol-3-glucoside (D3G), which was once regarded as a detoxification product of DON in plant defense, was detected when *Trichoderma* were confronted with *F. graminearum*. The occurrence of D3G in *F. graminearum* and *Trichoderma* interaction was reported for the first time, and these findings provide evidence that *Trichoderma* strains possess a self-protection mechanism as plants to detoxify DON into D3G when competing with *F. graminearum*.

## 1. Introduction

**Figure 1:**
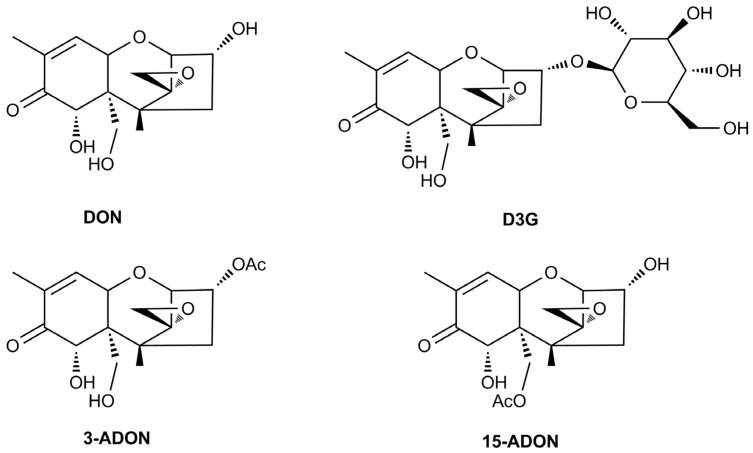
Chemical structures of deoxynivalenol (DON), deoxynivalenol-3-glucoside (D3G), and acetylated derivatives of DON: 3-acetyl-deoxynivalenol (3-ADON) and 15-acetyl-deoxynivalenol (15-ADON).

Mycotoxins are toxic secondary metabolites that are produced by toxigenic molds, and may contaminate different cereal grains [1]. Deoxynivalenol (DON), also known as vomitoxin, is mainly produced by the *Fusarium graminearum* species that can infect crops and cause devastating diseases called *Fusarium* head blight (FHB) or scab [2]. DON is an inhibitor of protein, DNA, and RNA synthesis at the molecular level, and exerts toxic potential to plants, animals, and humans [3]. The functions of DON in interactions of *F. graminearum* and other organisms have been studied before. As a crucial secondary metabolite in development of *F. graminearum*, DON is beneficial for *Fusarium* to deal with a complex environment and compete with other organisms [4,5]. *Fusarium* may utilize DON to disrupt plant defense system in the infection process. DON is regarded as a virulence factor when infecting plants, and it can facilitate disease spread in infected plant tissues. Genetic studies have proved that DON non-producing *Fusarium* mutants showed less virulence on crops than wild-type strains [6]. On the other hand, plants usually have the self-defense mechanisms to cope with mycotoxins, such as conjugating them with endogenous metabolites to less toxic products. An important detoxification process reducing the toxicity of DON in plants is glycosylation catalyzed by a special kind of glycosyltransferases [7], and a common detoxification product of DON in this reaction is deoxynivalenol-3-glucoside (D3G) (Figure 1). This conjugated mycotoxin was originally termed as a masked mycotoxin, because its structure has been changed and it may escape routine detection by conventional analytical methods [8].

The pathogens causing FHB are also capable of producing 3-acetyl-deoxynivalenol (3-ADON) or 15-acetyl-deoxynivalenol (15-ADON), which are commonly-detected acetylated derivatives of DON in contaminated grains or food commodities [2]. Both DON and ADONs belong to B-type trichothecene mycotoxins (Figure 1) characterized by a keto-group at position C-8 in their molecule structures [2]. The epoxide group is considered as a key factor determining the toxicity of trichothecene mycotoxins [9], and ADONs also possess toxic effects [10,11]. In order to protect consumers, DON and its acetylated types have all been included in the group provisional maximum tolerable daily intake (PMTDI) according to the maximum tolerated levels of DON in food enacted by the Joint FAO/WHO Expert Committee on Food Additives (JECFA) [10].

To reduce the risks caused by DON contamination in the food chain, plenty of measures, including selection of resistant cultivars, application of fungicide, and biological control agents (BCAs), have been studied and used to control DON-producing pathogens [12,13]. Among these mentioned strategies, biological control emerging as a green approach has captured significant attention recently. Natural fungicides, antagonistic microorganisms, and detoxification enzymes are the most-concerned functional BCAs to control DON contamination [14]. Antagonistic microorganisms could inhibit the development and mycotoxin production of toxigenic pathogens. *Trichoderma* strains, *Bacillus* strains, *Clonostachys rosea*, and *Cladosporium cladosporioides* are promising antagonistic microorganisms to manage DON contamination [15,16]. Among them, *Trichoderma* strains have been widely investigated and applied as beneficial BCAs in agriculture. They can protect crops against plant pathogens environment-friendly, and biological control activity of *Trichoderma* is mainly due to antibiotic production to inhibit the growth of pathogens, substrate competition to restrict colony areas of pathogens, and the ability to activate plant defense responses against pathogens [17,18].

The aim of our work was to study the potential antagonistic activities of selected *Trichoderma* strains for management of *F. graminearum* by dual culture assay. We assessed the anti-growth activity of the different *Trichoderma* strains on *F. graminearum*. Meanwhile, DON and its acetylated or glycosylated forms were monitored to evaluate the anti-toxigenic activity of antagonists. Interestingly, the modified mycotoxin D3G, which was previously regarded as a detoxification product in plant defense against DON-producing *Fusarium* [7], was detected when *F. graminearum* was co-cultured with *Trichoderma* strains. To our knowledge, this is the first time that it has been reported that the D3G is a detoxification product of *Trichoderma* when in competition with *F. graminearum*, which may provide new understandings on the interaction of antagonistic microbes and toxigenic *Fusarium*.

## 2. Results

### 2.1. Impact of Trichoderma Strains on Growth of F. graminearum 5035 in Dual Culture

**Figure 2:**
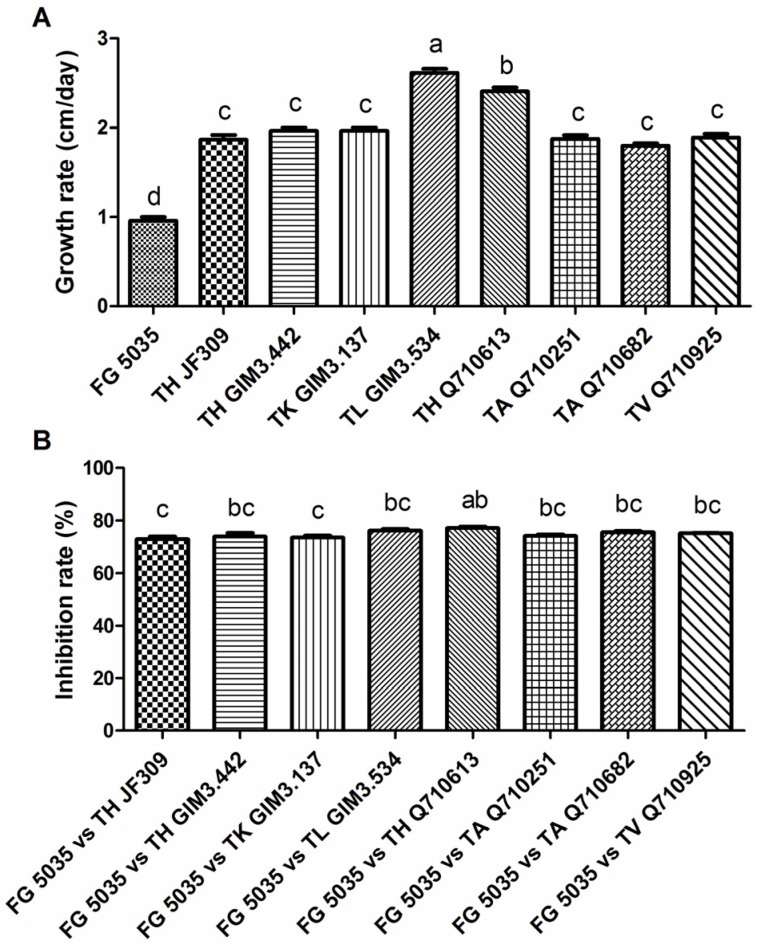
Growth rate (cm/day) of *F. graminearum* 5035 and *Trichoderma* strains (**A**). From left to right: the growth rate of *F. graminearum* 5035, *T. harzianum* JF309, *T. harzianum* GIM3.442, *T. koningii* GIM3.137, *T. longibranchiatum* GIM3.534, *T. harzianum* Q710613, *T. atroviride* Q710251, *T. asperellum* Q710682, and *T. virens* Q710925, respectively. Inhibition rate (%) of *Trichoderma* strains on mycelial growth of *F. graminearum* 5035 in dual culture (**B**). From left to right: *F. graminearum* 5035 grew against *T. harzianum* JF309, *T. harzianum* GIM3.442, *T. koningii* GIM3.137, *T. longibranchiatum* GIM3.534, *T. harzianum* Q710613, *T. atroviride* Q710251, *T. asperellum* Q710682, and *T. virens* Q710925, respectively. Data were from two independent experiments and shown as the mean ± SEM. Bars with different letters were significantly different according Tukey’s Test (α < 0.05) following one-way ANOVA (analysis of variance) analysis.

*Trichoderma* is recognized as a non-pathogenic genus majorly found in soil and plants, and has been widely studied due to its biological control-related characteristics [19]. In our antagonistic assay, the growth rates of selected *Trichoderma* strains were much faster than that of *F. graminearum* 5035 (Figure 2A). The mycelia of tested fungi began to contact two days after incubation. The faster growth rate made *Trichoderma* quickly occupy the living space and surround the colony of *Fusarium*, and then *Trichoderma* effectively suppressed the mycelial growth of *Fusarium*. The inhibition rate of *Trichoderma* on mycelial growth of *Fusarium* ranged from 73% to 77% (Figure 2B). For the pathogen, it was hard to overgrow antagonists for further extension on PDA medium.

**Figure 3:**
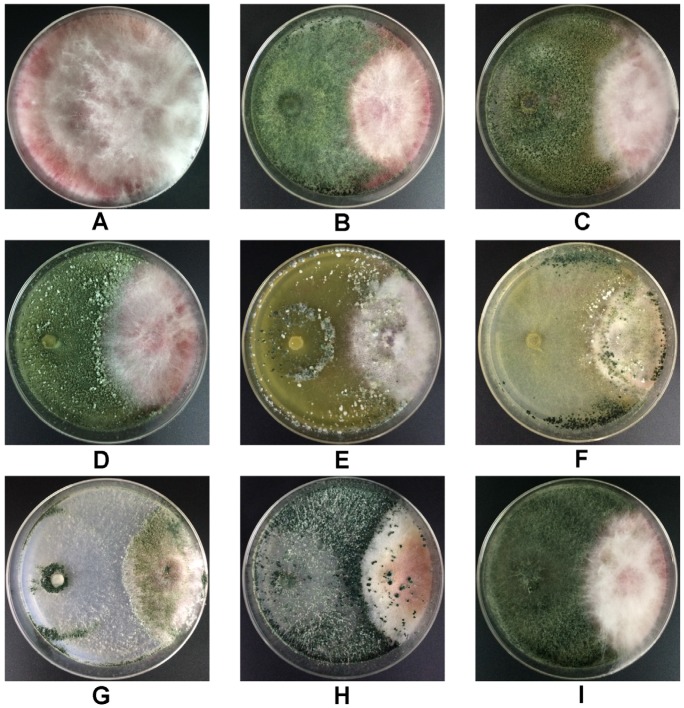
Colony morphology of *F. graminearum* 5035 in dual culture tests after incubation on the potato dextrose agar (PDA) medium. *F. graminearum* 5035 grew alone (**A**); *F. graminearum* 5035 grew against *T. harzianum* JF309 (**B**); *T. harzianum* GIM3.442 (**C**); *T. koningii* GIM3.137 (**D**); *T. longibranchiatum* GIM3.534 (**E**); *T. harzianum* Q710613 (**F**); *T. atroviride* Q710251 (**G**); *T. asperellum* Q710682 (**H**); and *T. virens* Q710925 (**I**).

Moreover, we observed the colony morphology of *Fusarium* when alone or in the presence of antagonists after incubation (Figure 3), finding that four *Trichoderma* strains including *T. longibranchiatum* GIM 3.534, *T. harzianum* Q710613, *T. atroviride* Q710251 and *T. asperellum* Q710682 were able to overgrow and sporulate on the colony of *F. graminearum* 5035, and the mycelia of *Fusarium* in these combinations were relatively flat, while the mycelia of *Fusarium* in other combinations were much denser. These signs demonstrated that the four *Trichoderma* strains capable of overgrowing the pathogen were more effective in inhibiting the mycelia spread of *Fusarium* on PDA medium.

*Trichoderma* strains have a stronger capacity to occupy living space and take up nutrients than other microbes [20]. It seems that growth suppression is the major inhibition pattern of *Trichoderma* when in competition with pathogens.

### 2.2. Effect of Trichoderma Strains on Mycotoxin Production of F. graminearum 5035 in Dual Culture

DON-producing *Fusarium* could infect crops in field and subsequently cause DON accumulation in cereal grains. Prevention before harvest is an important strategy to manage DON contamination, and antagonistic *Trichoderma* strains are promising BCAs to achieve this goal [21]. Matarese et al. has revealed that a *Trichoderma gamsii* strain 6085 could strongly reduce DON production of *Fusarium* on rice medium [17]. In order to accurately reveal the effect of *Trichoderma* strains on mycotoxin production of *F. graminearum*, DON and ADONs were all monitored by a liquid chromatography tandem mass spectrometry (LC-MS/MS) method in our work.

**Figure 4:**
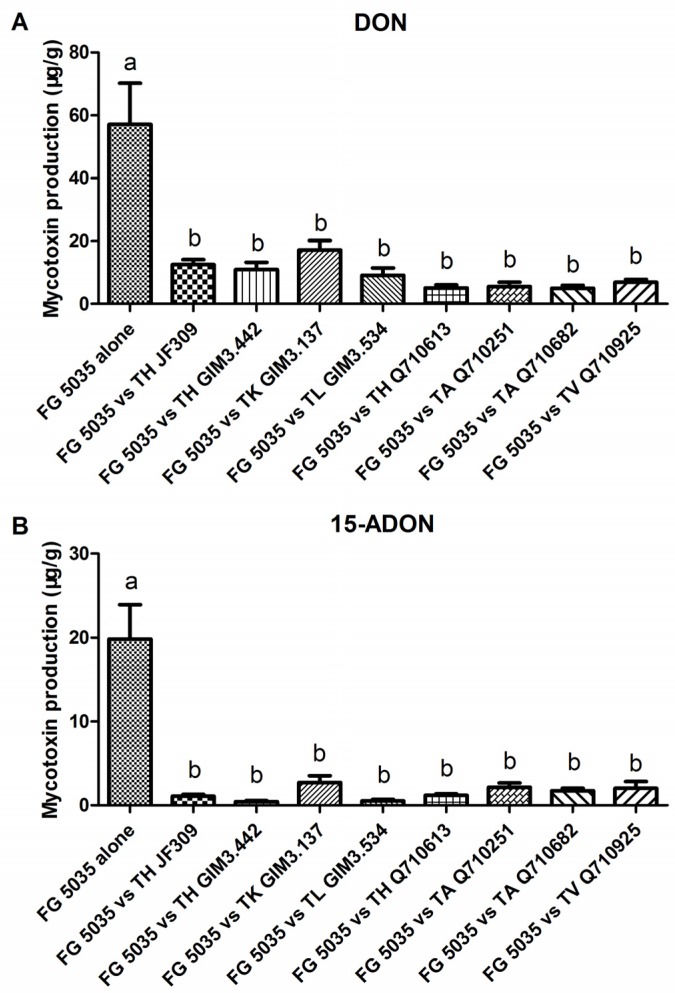
Mycotoxin production (DON and 15-ADON) of *F. graminearum* 5035 when alone or against different *Trichoderma* strains. From left to right: *F. graminearum* 5035 grew alone, and grew against *T. harzianum* JF309, *T. harzianum* GIM3.442, *T. koningii* GIM3.137, *T. longibranchiatum* GIM3.534, *T. harzianum* Q710613, *T. atroviride* Q710251, *T. asperellum* Q710682 and *T. virens* Q710925 (**A**,**B**). Data were from two independent experiments and shown as the mean ± SEM. Bars with different letters were significantly different according Tukey’s Test (α < 0.05) following one-way ANOVA analysis.

The data showed that *F. graminearum* 5035 was able to produce 57 μg/g DON and 20 μg/g 15-ADON on PDA medium when alone, indicating that the tested *F. graminearum* was a 15-ADON producer mainly producing DON and 15-ADON [2]. When *F. graminearum* 5035 was co-cultured with *Trichoderma*, the amount of DON produced by *F. graminearum* reduced significantly due to the strong antagonistic action of *Trichoderma*. When in the presence of *T. harzianum* Q710613, *T. atroviride* Q710251 and *T. asperellum* Q710682, the amount of DON produced by *F. graminearum* 5035 decreased more than 90% compared with the control. In the presence of other *Trichoderma* strains, the inhibition rate of DON production ranged from 70% to 88% (Figure 4A). With regard to 15-ADON, the antagonists seemed to exhibit stronger inhibition capacity: the inhibition rate of 15-ADON production ranged from 86% to 98% (Figure 4B). In consideration of the non-ignorable toxicity of ADONs [22], DON and its acetylated forms should be all monitored when assessing the anti-toxigenic activity of antagonistic microbes.

### 2.3. Occurrence of Modified Mycotoxin D3G in Dual Culture of Trichoderma and F. graminearum

Modified mycotoxin D3G is the most prevalent detoxification product of DON in self-defense of plants, and detoxification via glycosylation is very general in plants. However, this biotransformation process is unusual in microbes [23]. A previous study has emphasized that DON production of *F. graminearum* could not reduce the biomass of *Trichoderma* when in competition [24]. Therefore, we speculated that *Trichoderma* strains were very likely to possess some underlying detoxification mechanisms against DON-producing *F. graminearum*. In order to verify whether *Trichoderma* strains could detoxify DON via glycosylation when confronted with *F. graminearum*, D3G was monitored in our work.

**Figure 5:**
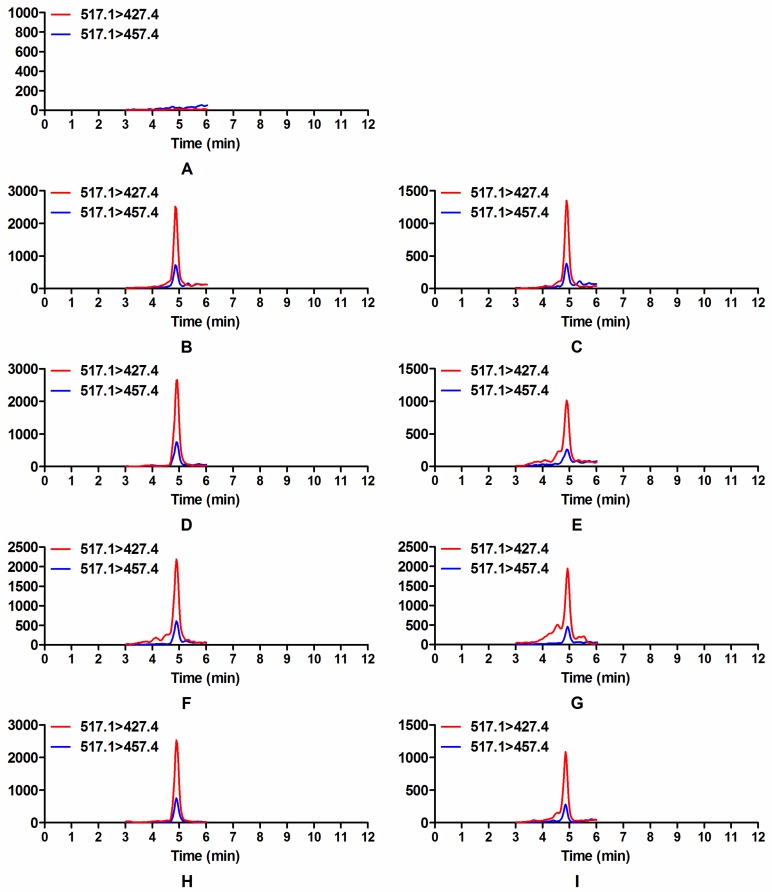
Selected reaction monitoring (SRM) chromatograms of D3G in samples of dual culture test: *F. graminearum* 5035 grew against *T. harzianum* JF309 (**B**); *T. harzianum* GIM3.442 (**C**); *T. koningii* GIM3.137 (**D**); *T. longibranchiatum* GIM3.534 (**E**); *T. harzianum* Q710613 (**F**); *T. atroviride* Q710251 (**G**); *T. asperellum* Q710682 (**H**); and *T. virens* Q710925 (**I**). Red line (*m*/*z* 517.1 > *m*/*z* 427.4) and blue line (*m*/*z* 517.1 > *m*/*z* 457.4) show the SRM traces of D3G. There was no D3G detected when *F. graminearum* 5035 grew alone on PDA medium (**A**).

Based on our LC-MS/MS method, the glycosylation form of DON (D3G), was detected when *F. graminearum* 5035 was co-cultured with *Trichoderma* strains, while there was no D3G detected in the control group (Figure 5). Glycosylation of DON catalyzed by UDP-glucosyltransferases (UGTs) is a well-known detoxification process in plants, and the first UGT capable of transforming DON to D3G was identified from *Arabidopsis thaliana* [7]. However, no studies have reported the occurrence of modified mycotoxin D3G in interactions between microbes so far. In order to confirm the presence of D3G in the co-culture of *Trichoderma* and *F. graminearum*, prepared samples of dual culture assay were analyzed by liquid chromatography high resolution mass spectrometry (LC-HRMS). Shown as in Figure S1, the MS/MS spectra of the precursor ion (*m*/*z* 517.1927, [M + Ac]^−^) in samples of the dual culture test were in good agreement with the MS/MS spectra of D3G standard. The key fragments *m*/*z* 427.16, 457.17, 409.15, 277.11, 247.10 for [D3G + Ac]^−^ were also observed in the previous study [25]. These results provided evidences for the existence of D3G in dual cultures of *Trichoderma* and DON-producing *Fusarium*. The occurrence of D3G in *Trichoderma* and *Fusarium* interaction indicated that D3G was a detoxification product in self-defense of *Trichoderma* against DON-producing *Fusarium*.

Our preliminary results provide evidence that *Trichoderma* strains not only possess the strong inhibitory action on *F. graminearum*, but also possess the detoxification capability to glycosylate DON when competing with *F. graminearum*. All of these advantageous features make *Trichoderma* strains promising BCAs against plant disease in agriculture.

### 2.4. A Significant Difference in D3G/DON Ratios of Trichoderma Strains when Confronted with F. graminearum 5035

**Figure 6:**
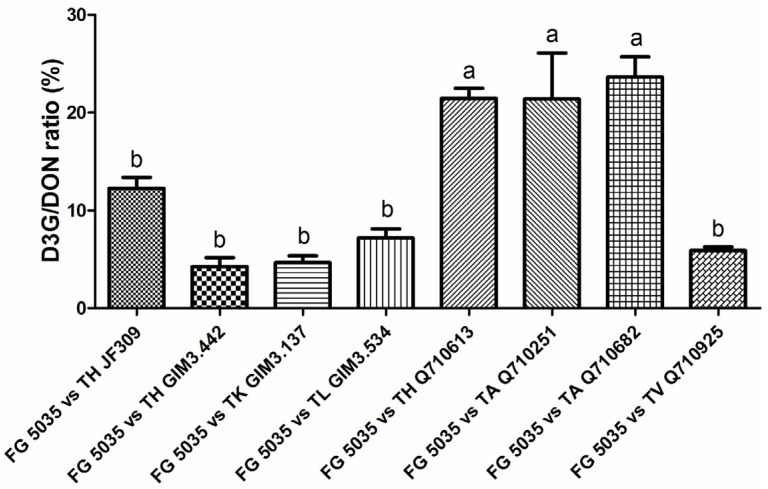
D3G/DON ratios of different *Trichoderma* strains when confronted with *F. graminearum* 5035 in dual culture. *F. graminearum* 5035 grew against *T. harzianum* JF309, *T. harzianum* GIM3.442, *T. koningii* GIM3.137, *T. longibranchiatum* GIM3.534, *T. harzianum* Q710613, *T. atroviride* Q710251, *T. asperellum* Q710682; and *T. virens* Q710925, respectively. Data were from two independent experiments and shown as the mean ± SEM. Bars with different letters were significantly different according Tukey’s Test (α < 0.05) following one-way ANOVA analysis.

Previous studies regarding pathogen and plant interactions have illustrated that D3G/DON ratios of various wheat lines can be regarded as their resistance indicators against FHB [8,26,27]. Similarly, D3G/DON ratios of different *Trichoderma* strains used in this study may also be related to their resistance to *Fusarium*. In our work, we assessed the D3G/DON ratios of tested *Trichoderma* strains when dual cultured with DON-producing *F. graminearum* 5035. Interestingly, the D3G/DON ratios of different *Trichoderma* strains that might reflect their resistance to *Fusarium* were significantly different (Figure 6). *T. harzianum* Q710613, *T. atroviride* Q710251 and *T. asperellum* Q710682 showed relatively higher ratios that exceeded 20%, while D3G/DON ratios of other *Trichoderma* strains were relatively lower, which were in the range of 4% to 12%. It seems that DON-resistant antagonists have more potential in further investigation and application, because recent studies have showed that over-expression of UGT genes capable of detoxifying DON into D3G in crops could improve their resistance against *Fusarium* [28,29], and the reported functional UGT genes were all derived from plants. Thus, our data revealed that *Trichoderma* might be a potential source to seek efficient UGT genes, which can be expressed to detoxify DON directly or be genetically-engineered in crops to improve their resistance against FHB causal agents [30].

Understanding the mechanisms of the antagonistic action of BCAs on pathogens is essential for searching potential *Trichoderma* strains to manage plant disease. Lutz et al. suggested that the role of DON in competitiveness of *F. graminearum* was a negative signal, which down-regulated one chitinase gene (*nag1*) involved in biological control activity of an antagonistic *T. atroviride* strain [31]. However, our study provided evidence for a new role of DON in the competition of *Trichoderma* and *Fusarium*: DON not only modulated biological-control related genes of *Trichoderma*, but also induced *Trichoderma* self-defense related genes to detoxify mycotoxin DON. The responses of *Trichoderma* to DON-producing *F. graminearum* 5035 were different according to the D3G/DON ratios, which may represent their resistance against DON-producing pathogens, and this hypothesis needs to be validated in future work. Generally, the metabolic fate of DON in *Trichoderma* needs more detailed study to present new antagonistic mechanisms of *Trichoderma*.

## 3. Discussion

FHB caused by *F. graminearum* species could reduce the yield and quality of cereal grains around the world, and DON contamination in the food chain leads to deleterious effects on the health of animals and humans [12]. Biological control of *F. graminearum* and mycotoxin DON contamination would be an important part in integrated FHB management, which may avoid negative effects and minimize environmental impacts caused by conventional approaches [15]. Application of *Trichoderma* strains on crops or crop residuals to inhibit the development and DON production of *Fusarium* is an available biological-based strategy. As potential BCAs, *Trichoderma* strains are capable of producing a large number of enzymes and secondary metabolites, which are effective components against pathogens and play an important role in the antagonistic action of *Trichoderma* [32]. Hydrolytic enzymes (such as chitinase and proteases) and antibiotics (such as polyketides and terpenes) derived from *Trichoderma* act as “weapons” against pathogens when in competition [32]. In addition, the potential UGTs inactivating DON in *Trichoderma* strains, act as “shields” to protect themselves against DON-producing pathogens. For *Trichoderma*, “weapons” and “shields” are all significant agents for control of *Fusarium*. For plants, glycosylation of DON catalyzed by the UGTs is an effective defense strategy to resist pathogens [27], and our present study indicates that *Trichoderma* also have a defense capacity to detoxify DON as plants when against DON-producing *Fusarium*. Our findings are supported by the detection of modified mycotoxin D3G in a dual culture of *Trichoderma* and *Fusarium*. Suzuki et al. have already elucidated that D3G exhibited extremely low toxicity to yeast and algae, indicating that detoxification via glycosylation may not be exclusive to plants [33]. Moreover, our data confirmed that glycosylation of DON was a self-defense process for *Trichoderma* as well, which is in accordance with a previous study of Suzuki et al. A novel understanding on the role of DON in the competitiveness of *Fusarium* would promote deeper investigation of the interaction between antagonists and toxigenic *Fusarium* species.

The metabolism of mycotoxins in plants has been studied previously. Plants have the detoxification metabolic systems to counter phytotoxic compounds. Mycotoxins can be conjugated to endogenous metabolites by plants, generating modified mycotoxins with decreased toxicity [27]. In plants, detoxification of mycotoxins usually includes three phases. In phase I, mycotoxins are oxidized or hydrolyzed to generate some active sites for the next reaction. In phase II, low-toxic or non-toxic metabolites are formed by conjugating some hydrophilic molecules, such as sugars or amino acids, and D3G is the major metabolite of DON in this phase. Finally, the metabolites from phase II are irreversibly transported to a vacuole or accumulated in the cell wall in phase III [8]. Glycosylation catalyzed by UGTs is a major process of phase II in detoxification reactions of plants [33]. A recent study has clearly validated that over-expressing such UGT genes able to detoxify DON in *Brachypodium* could confer increased resistance to *Fusarium* [34]. Glycosyltransferases are a series of enzymes that can catalyze the formation of a glycosidic bond by transferring sugars to substrates, such as plant secondary metabolites and mycotoxins. Identification of the UGTs inactivating DON into D3G is rather complex because UGTs are encoded by a large family: more than 100 putative gene members in plants [35]. To date, some induced functional UGT genes during the DON-producing *Fusarium* infection process have been studied and identified in different plants [28,34,35,36,37,38]. However, little is known about such UGTs possessing DON-detoxification ability in *Trichoderma* strains. According to previous studies, *Trichoderma* can produce non-phytotoxic trichothecenes, such as trichodermin and harzianum [18,39], but the mechanism of DON metabolism in *Trichoderma* strains is unclear. Taken together, the studies on identifying these specific UGT genes from *Trichoderma* strains by transcriptome analysis of *Trichoderma* and DON-producing *Fusarium* interaction should be conducted in the future. The details about the endogenous self-protection mechanism against DON in *Trichoderma* strains needs further investigation and interpretation after the detoxification UGT genes from *Trichoderma* are identified. On the other hand, other detoxification products of DON have been identified in plant detoxification processes, such as DON-sulfates, DON-15-glucoside, and DON-glutathione [25,40,41]. Seeking and identifying other modified mycotoxins generated from *Trichoderma* and *Fusarium* are also essential to reveal the mycotoxin detoxification mechanism of *Trichoderma*.

## 4. Conclusions

In the present study, eight *Trichoderma* strains were selected to evaluate their antagonistic activities against *F. graminearum* by dual culture assay. Results suggested that *T. harzianum* Q710613, *T. atroviride* Q710251, and *T. asperellum* Q710682 were more effective antagonists to control the pathogen, not only because of the efficacy in inhibiting mycelia spread and mycotoxin production, but also because of the relatively higher D3G/DON ratios that may reflect their resistance to *Fusarium*. In addition, the occurrence of modified mycotoxin D3G in a dual culture of *Fusarium* and *Trichoderma* provides new insights into antagonistic activities of *Trichoderma*.

## 5. Materials and Methods

### 5.1. Standards and Chemicals

The mycotoxin standards of DON, D3G, 3-ADON, and 15-ADON were purchased from Sigma-Aldrich (St. Louis, MO, USA). Methanol and acetonitrile (ACN) were all high performance liquid chromatography(HPLC) grade, and obtained from Merck (Darmstadt, Germany). Other solvents and chemicals of HPLC or analytical grade were provided by Aladdin (Shanghai, China). Ultrapure water (18.2 MΩ·cm) was obtained from Millipore (Bedford, MA, USA), and used throughout the experiments.

### 5.2. Fungal Strains and Culture Medium

A total of eight *Trichoderma* strains and one *F. graminearum* strain were used in the present study (Table 1). *F. graminearum* 5035 was from the Huazhong Agricultural University [42]. *T. harzianum* JF309 was isolated from *Lentinus edodes* in our lab. *T. harzianum* GIM3.442, *T. koningii* GIM3.137, and *T. longibranchiatum* GIM3.534 were purchased from the Microbial Culture Collection Center of Guangdong Institute of Microbiology (GIMCC). *T. harzianum* Q710613, *T. atroviride* Q710251, *T. asperellum* Q710682, and *T. virens* Q710925 were from the Center for Culture Collection of *Trichoderma* (CCCT), Shanghai Jiaotong University (SJTU). All of the fungal strains were maintained as spore suspensions in 30% glycerol at −80 °C. Before the start of antagonistic assay, the fungal strains were grown on PDA medium at 25 °C for five days for activation.

### 5.3. Antagonistic Activities of Trichoderma on Growth and Mycotoxin Production of F. graminearum

The antagonistic activities of the chosen *Trichoderma* strains on *F. graminearum* were assessed by dual culture assay according to Matarese et al. [17] with minor modifications. In short, 6 mm-diameter mycelial discs of tested *Trichoderma* and *Fusarium* strains were removed from the edge of actively-growing colonies, and then the mycelial discs (*Fusarium* and *Trichoderma* combinations) were placed at a distance of 45 mm on a 90 mm-diameter Petri dish containing 20 mL PDA medium. Meanwhile, a mycelial disc of *F. graminearum* 5035 was placed with an agar disc on the dish as the control. The pathogen-antagonist combinations and the controls were all set up in three biological replicates, and incubated at 25 °C for 14 days. The mycelial growth rates (cm/day) of *Trichoderma* strains and *F. graminearum* were recorded before their mycelia began to interact. After incubation, the radii of *Fusarium* colonies facing the antagonists were measured and used for calculation of the inhibition rate [32]. The inhibition rate of *Trichoderma* on mycelial growth of tested *F. graminearum* was calculated based on the following formula: *I* = (*C* − *T*)/*C* × 100, where *I* is the inhibition rate (%), *C* is the growth radius of pathogen when alone, and *T* is the growth radius of the pathogen when co-cultured with antagonists [32].

### 5.4. Fusarium Mycotoxin Extraction and Preparation

After incubation, the PDA medium containing mycelia of tested fungi strains in Petri dish was dried until a constant weight, and then the sample was ground into a homogenized powder for preparation as described previously [43].

The homogenized powder was weighed and put into a 50 mL centrifuge tube, and then 10 mL of ACN/water (84/16, *v*/*v*) solution was added. The mixture was shaken for 5 min, and then subjected to ultrasonication for 30 min. Next the mixture was centrifuged at 3200× *g* for 30 min. Then 2 mL of supernatant was transferred into a centrifuge tube, followed by adding 150 mg of MgSO_4_. The mixture was vortexed for 30 s, and centrifuged at 3200× *g* for 30 min. Thereafter, the remaining supernatant was moved into a new tube, and evaporated to dryness by a stream of nitrogen gas at 40 °C. At last, the residue was redissolved with 1 mL of water containing 5 mM ammonium acetate. After redissolution, the mixture was passed through a 0.22-μm filter, and then injected into LC-MS/MS for analysis.

### 5.5. Mycotoxin Determination by LC-MS/MS

Mycotoxin concentrations were determined according to a published method with minor modifications [43]. Briefly, chromatographic separation was performed on an Agilent Extend—C18 column (100 mm × 4.6 mm, 3.5 µm) at 30 °C using a Thermo Scientific Accela 1250 UPLC system (Thermo Fisher Scientific, San Jose, CA, USA). The mobile phase consisted of water containing 5 mM ammonium acetate (A) and methanol (B), and a gradient elution program was as follows: 0 min 15% B, 1 min 15% B, 6.5 min 90% B, 8.5 min 90% B, 9 min 15% B, 12 min 15% B. The injection volume was 10 μL and the flow rate was 0.35 mL/min.

MS/MS analysis was carried out using a Thermo Scientific TSQ VantageTM (Thermo Fisher Scientific, San Jose, CA, USA) triple stage quadrupole mass spectrometer in both positive and negative electrospray ionization (ESI^+^/ESI^−^) modes. The ionization source parameters were set as follows: interface voltage of 3.5 kV (ESI^+^) or 3.0 kV (ESI^−^); desolvation line (DL) temperature of 250 °C; nebulizing gas (N_2_) and drying gas (N_2_) pressure of 30 psi (207 kPa) and 20 psi (138 kPa), respectively; heat block temperature of 350 °C. A series of standard solutions of analytes (10–1000 ng/mL) were used for calibration to absolutely quantify the targeted mycotoxins in the samples, and the quantitation and identification of target mycotoxins were performed in selected reaction monitoring (SRM) mode. The MS/MS parameters of detected mycotoxins were summarized in Table 2. All data acquisition and processing were achieved with Xcalibur™ Software (2.2, Thermo Fisher Scientific, San Jose, CA, USA, 2011).

### 5.6. LC-HRMS Analysis of D3G

LC–HRMS analysis of the fragmentation pattern of D3G was conducted on a UHPLC system (1290 series, Agilent Technologies, Santa Clara, CA, USA) coupled to a quadruple time-of-flight (Q-TOF) mass spectrometer (Agilent 6550 iFunnel Q-TOF, Agilent Technologies, Santa Clara, CA, USA). Chromatographic separation was performed on an Agilent Extend—C18 column (100 mm × 4.6 mm, 3.5 µm) at 30 °C. The mobile phase consisted of water containing 5 mM ammonium acetate (A) and methanol (B), and a gradient elution program was as follows: 0 min 15% B, 1 min 15% B, 6.5 min 90% B, 8.5 min 90% B, 9 min 15% B, and 12 min 15% B. The flow rate was 0.35 mL/min and the sample injection volume was 5 μL.

To confirm the presence of D3G in samples of dual culture test, the negative precursor ion *m*/*z* 517.1927 (theoretical mass of [D3G + Ac]^−^ based on molecular formula) was ionized in an electrospray ionization (ESI) source and mass isolated by quadruple mass filter, following by collision-induced dissociation (CID) with the collision energy of −20 eV and, finally, the fragments and the remaining precursor were detected in the TOF tube. The parameters of high resolution mass spectrometer were set as follows: sheath gas (N_2_) temperature, 300 °C; dry gas (N_2_) temperature, 170 °C; sheath gas flow, 12 L/min; dry gas flow, 16 L/min; capillary voltage, 3.0 kV in negative mode; nozzle voltage, 0 V; fragmentor, 175 V; and nebulizer pressure, 40 psi (276 kPa). Data analysis was achieved with Agilent software MassHunter B.06.00 (Agilent Technologies, Santa Clara, CA, USA, 2012).

### 5.7. Statistical Analysis

Data were subjected to one-way ANOVA (analysis of variance) analysis followed by Tukey’s multiple comparisons test using Graphpad Prism 5.0 (GraphPad Software, San Diego, CA, USA, 2007). All data were shown as the mean ± standard error of the mean (SEM), and *p* < 0.05 was considered to be significantly different.

## Figures and Tables

**Table 1:** The information of the fungal strains used in this study.

Strain Code	Strain	Source
5035	*Fusarium graminearum*	Huazhong Agricultural University, China
JF309	*Trichoderma harzianum*	Isolated from *Lentinus edodes* in our lab
GIM3.442	*Trichoderma harzianum*	GIMCC, China
GIM3.137	*Trichoderma koningii*	GIMCC, China
GIM3.534	*Trichoderma longibranchiatum*	GIMCC, China
Q710613	*Trichoderma harzianum*	CCCT, SJTU, China
Q710251	*Trichoderma atroviride*	CCCT, SJTU, China
Q710682	*Trichoderma asperellum*	CCCT, SJTU, China
Q710925	*Trichoderma virens*	CCCT, SJTU, China

GIMCC: Microbial Culture Collection Center of Guangdong Institute of Microbiology; CCCT, SJTU: Center for Culture Collection of *Trichoderma*, Shanghai Jiaotong University.

**Table 2:** The tandem mass spectrometry (MS/MS) parameters for target mycotoxins in selected reaction monitoring (SRM) mode.

Mycotoxin	Precursor Ion (*m*/*z*)	Retention Time (min)	Primary Product Ion (*m*/*z*)	Collision Energy (ev)	Secondary Product Ion (*m*/*z*)	Collision Energy (ev)
DON	355.1 [M + Ac]^−^	5.04	265.0 ^a^	17	247.2 ^b^	18
D3G	517.1 [M + Ac]^−^	4.89	427.4 ^a^	22	457.4 ^b^	14
3-ADON	397.1 [M + Ac]^−^	6.54	307.1 ^a^	16	173.1 ^b^	15
15-ADON	356.1 [M + NH_4_]^+^	6.52	321.0 ^a^	15	137.0 ^b^	12

^a^ Ion for quantification; ^b^ Ion for identification.

## Supplementary Materials

The following are available online at www.mdpi.com/2072-6651/8/11/335/s1, Figure S1: Tandem mass spectra of the precursor ion (*m*/*z* 517.1927, [M + Ac]^−^) in negative mode. (A) Product ion spectra of the D3G standard (1000 ng/mL); (B–I) product ion spectra of the precursor ion (*m*/*z* 517.1927) with the same chromatographic retention time as the D3G standard in samples of dual culture test: *F. graminearum* 5035 grew against *T. harzianum* JF309 (B); *T. harzianum* GIM3.442 (C); *T. koningii* GIM3.137 (D); *T. longibranchiatum* GIM3.534 (E); *T. harzianum* Q710613 (F); *T. atroviride* Q710251 (G); *T. asperellum* Q710682 (H); and *T. virens* Q710925 (I).
