# Supplementary material for: Detoxification of Deoxynivalenol via Glycosylation Represents Novel Insights on Antagonistic Activities of Trichoderma when Confronted with Fusarium graminearum
Source: Toxins (Basel). 2016 Nov 15;8(11):335. doi: 10.3390/toxins8110335 (PMC5127131; doi:10.3390/toxins8110335)
Supplement: Supplementary file 1 [file toxins-08-00335-s001.pdf]

Supplementary Materials: Detoxification of Deoxynivalenol via Glycosylation Represents Novel Insights on Antagonistic Activities of *Trichoderma* when Confronted with *Fusarium graminearum*

Ye Tian, Yanglan Tan, Na Liu, Zheng Yan, Yucai Liao, Jie Chen, Sarah de Saeger, Hua Yang, Qiaoyan Zhang and Aibo Wu

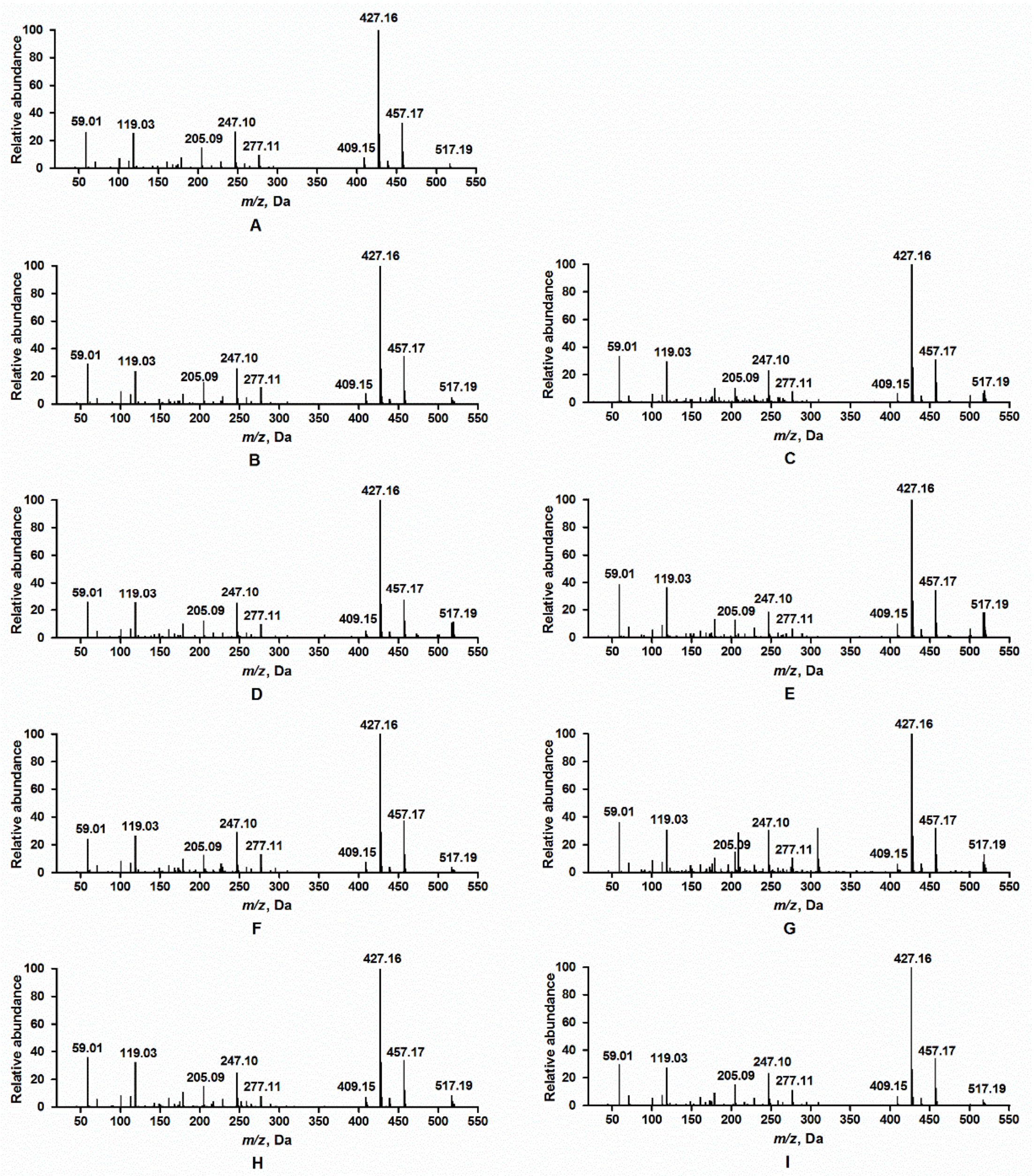

Figure S1. Tandem mass spectra of the precursor ion (*m*/*z* 517.1927, [M + Ac]$^-$) in negative mode. (**A**) Product ion spectra of the D3G standard (1000 ng/mL); (**B**–**I**) product ion spectra of the precursor ion (*m*/*z* 517.1927) with the same chromatographic retention time as the D3G standard in samples of dual culture test: *F. graminearum* 5035 grew against *T. harzianum* JF309 (**B**); *T. harzianum* GIM3.442 (**C**); *T. koningii* GIM3.137 (**D**); *T. longibranchiatum* GIM3.534 (**E**); *T. harzianum* Q710613 (**F**); *T. atroviride* Q710251 (**G**); *T. asperellum* Q710682 (**H**); and *T. virens* Q710925 (**I**).
